# A reference architecture for personal health data spaces using decentralized content-addressable storage networks

**DOI:** 10.3389/fmed.2024.1411013

**Published:** 2024-07-16

**Authors:** Toomas Klementi, Gunnar Piho, Peeter Ross

**Affiliations:** ^1^Department of Software Science, Tallinn University of Technology (TalTech), Tallinn, Estonia; ^2^Department of Health Technologies, TalTech, Tallinn, Estonia; ^3^Research Department, East Tallinn Central Hospital, Tallinn, Estonia

**Keywords:** health data accessibility, comprehensiveness, and ownership dilemmas, primary and secondary use, a reference architecture for global health data space, decentralized content-addressable storage (DCAS) networks, semantic interoperability, European Health Data Space (EHDS)

## Abstract

**Introduction:**

This paper addresses the dilemmas of accessibility, comprehensiveness, and ownership related to health data. To resolve these dilemmas, we propose and justify a novel, globally scalable reference architecture for a Personal Health Data Space (PHDS). This architecture leverages decentralized content-addressable storage (DCAS) networks, ensuring that the data subject retains complete control and ownership of their personal health data. In today's globalized world, where people are increasingly mobile for work and leisure, healthcare is transitioning from episodic symptom-based treatment toward continuity of care. The main aims of this are patient engagement, illness prevention, and active and healthy longevity. This shift, along with the secondary use of health data for societal benefit, has intensified the challenges associated with health data accessibility, comprehensiveness, and ownership.

**Method:**

The study is structured around four health data use case scenarios from the Estonian National Health Information System (EHIS): primary medical use, medical emergency use, secondary use, and personal use. We analyze these use cases from the perspectives of accessibility, comprehensiveness, and ownership. Additionally, we examine the security, privacy, and interoperability aspects of health data.

**Results:**

The proposed architectural solution allows individuals to consolidate all their health data into a unified Personal Health Record (PHR). This data can come from various healthcare institutions, mobile applications, medical devices for home use, and personal health notes.

**Discussions:**

The comprehensive PHR can then be shared with healthcare providers in a semantically interoperable manner, regardless of their location or the information systems they use. Furthermore, individuals maintain the autonomy to share, sell, or donate their anonymous or pseudonymous health data for secondary use with different systems worldwide. The proposed reference architecture aligns with the principles of the European Health Data Space (EHDS) initiative, enhancing health data management by providing a secure, cost-effective, and sustainable solution.

## 1 Introduction

Health data encompasses information about an individual's or a population's health conditions, health outcomes, and quality of life (1). They include clinical, environmental, socioeconomic, and behavioral data relevant to health and wellness (2). Healthcare digitalization, when combined with accurate and high-quality health data, presents opportunities for delivering enhanced health and wellness-related services at reduced costs (3). However, health data introduces significant risks, as alone or combined with other data, it can reveal personal health status (4). The risk of revealing health status may reduce the willingness of individuals to participate in certain care processes, e.g., in mental health (5, 6) or drug abuse treatment. Health data leakage can also lead to discrimination against individuals by employers, insurers, or banks (7, 8).

The primary use of health data for diagnosis, treatment, and rehabilitation expects that pertinent information about a person's health is shared accurately and promptly with relevant parties, facilitating coordinated decision-making across all care settings (9). Beyond primary use, health data is utilized for secondary purposes (10) by various stakeholders, including policymakers, public health officials, researchers, physicians, the public, and industry (11). Routine clinical data is considered highly valuable (12) for advancing healthcare objectives and improving overall health outcomes.

Despite the value of routine clinical data collected during healthcare provision, significant portions of health data remain underutilized (13) due to the unstructured nature of the data and privacy and interoperability concerns. Moreover, the integration of medical data from various health data sources—Electronic Health Records (EHRs), medical devices for home use, innovative health and welfare applications, and health notes by patients—is beneficial in both primary and secondary use (14). However, the challenges related to data security, privacy, accessibility, comprehensiveness, and interoperability (15) result in the underutilization of data integration. We formulate these challenges as the following three dilemmas.

*The dilemma of accessibility*: The conflict between the desire for the accessibility of health data and the need to safeguard sensitive personal information.

This dilemma encapsulates the contradiction between ensuring data FAIR accessibility (16) and protecting sensitive personal information (17). A vast dataset with valuable routine health data is available worldwide, and broad and open access to this information is essential to maximize its benefits for society and citizens (18). However, given the delicate nature of personal data, there's an increasingly pressing need to fortify access controls. This presents a notable contradiction, as the pursuit of widespread health data FAIR accessibility clashes with the imperative to protect personal information (19).

*The dilemma of comprehensiveness*: The challenge to reconcile the need for the comprehensiveness of health data with their current fragmented nature (20).

Currently, a person's health data are preserved in different service providers' data repositories in provider-specific formats, preventing the gathering of a holistic representation of the individual's health record (21). Using the complete personal health records of a person, modern machine learning and AI methods can be used to gain a comprehensive picture of their health status (22). This would enable a transition from episodic, symptom-based treatment to continuous health monitoring and personal integrated care pathways, aiming to prevent diseases or diagnose them as early as possible. However, various factors prevent consolidating an individual's health data into a single, unified repository, including challenges related to semantic interoperability, diverse legal and ethical hurdles, and elevated risks of data leakage. As stated in research from 2018 (23), we still do not have a unified interoperability approach to cope with the semantic heterogeneity of health data. A review from 2019 concludes that no big-data analytics will happen without optimized data sharing and reuse, which we still lack despite different interoperability standards in the medical domain (24). Similar semantic interoperability-related challenges will be highlighted in the papers published in 2024 (25, 26).

*The dilemma of ownership*: The discrepancy between the data owner's rights to ownership and the practical inability to exercise those rights.

The presented statement highlights a dual dilemma. First, whether data and information can be considered property remains unresolved (27, 28). Second, the significant challenges associated with data ownership need to be addressed. While this paper refrains from definitively answering the first question, the authors generally favor an affirmative stance. Regardless of the stance on data ownership, prevailing legislation (29) ensures specific rights for the data subject concerning the information collected about them. Generally, in the EU, the processing of health data is prohibited unless there is a lawful basis under Article 6 of the GDPR and one of the exceptions mentioned in Article 9 is met (e.g., consent, contract, legal obligation, vital interests, public tasks, and legitimate interest). This legal framework ensures that individuals maintain control over their health data, emphasizing the importance of informed consent and transparency in processing such data (30). In reality, however, the practical exercise of these rights faces challenges, as data is preserved in third-party servers beyond the physical control of the data subject. In most countries, laws governing medical records place responsibility for storing health data on healthcare providers. These regulations are based on the healthcare provision legislation and do not need to be discussed in the context of this article.

Even the contemporary regional or national digital health platforms (DHPs) like the Estonian National Health Information System (EHIS) cannot resolve these dilemmas. First, as such systems are data processors according to the GDPR, they must process, protect, and secure data accordingly. Therefore, accessing data for secondary purposes is difficult due to complex content management and the need for de-identification (anonymization and pseudonymization) (31). Second, in such systems, the dilemma of data comprehensiveness has not been solved because of the international mobility of citizens. To solve this, the DHP must be pan-European or worldwide, or there is a need for an interoperability solution for the federation of national health systems. This is likely impossible and impractical as such systems are too complex to develop and operate. The third challenge involves the data ownership dilemma. Within the intricate infrastructure of national or regional DHPs where data may be stored either in the cloud or on local servers, individuals do not know the whereabouts of their data. More critically, they might be unaware of who has access to their data and for what purposes it is being used. This situation further complicates individuals' ability to exercise their legal rights, leaving them powerless and disconnected from their health data.

In addition, the solution used in Estonia, which has 1.3 million citizens, may not be scalable in larger countries or, for instance, on a pan-European scale due to development and operation costs and data security and privacy challenges. One of the issues in such extensive DHP systems is health data concentration (32), which may be tempting for attackers because, in the event of a successful attack, it is possible to obtain the health data of many people. Between 2009 and 2022, there were 5,150 healthcare data breaches, resulting in the impermissible disclosure of 382,262,109 healthcare records in total (33). In 2021 alone, there were 686 HIPAA rule breaches affecting 500 or more health records, and the Accellion FTA Hack alone exposed the health information of at least 3.51 million individuals, making it the worst year for healthcare data breaches (34).

The more concentrated the data, the higher the costs for security; any breach could have severe consequences for individuals' privacy and well-being. Moreover, the dominance of a few entities in controlling health data raises questions about data ownership and control and the risks for data monopoly. Additionally, there are worries about the impact on healthcare innovation. A concentrated health data environment may hinder the development of diverse and competitive solutions, limiting the ability of small players to enter the market. Striking a balance between centralized and decentralized approaches, and prioritizing privacy and competition, is crucial in addressing the health data concentration issue. Policymakers, healthcare providers, and technology companies must collaborate on patient privacy, promote fair competition, and foster innovation in the health data ecosystem.

**Figure 1:**
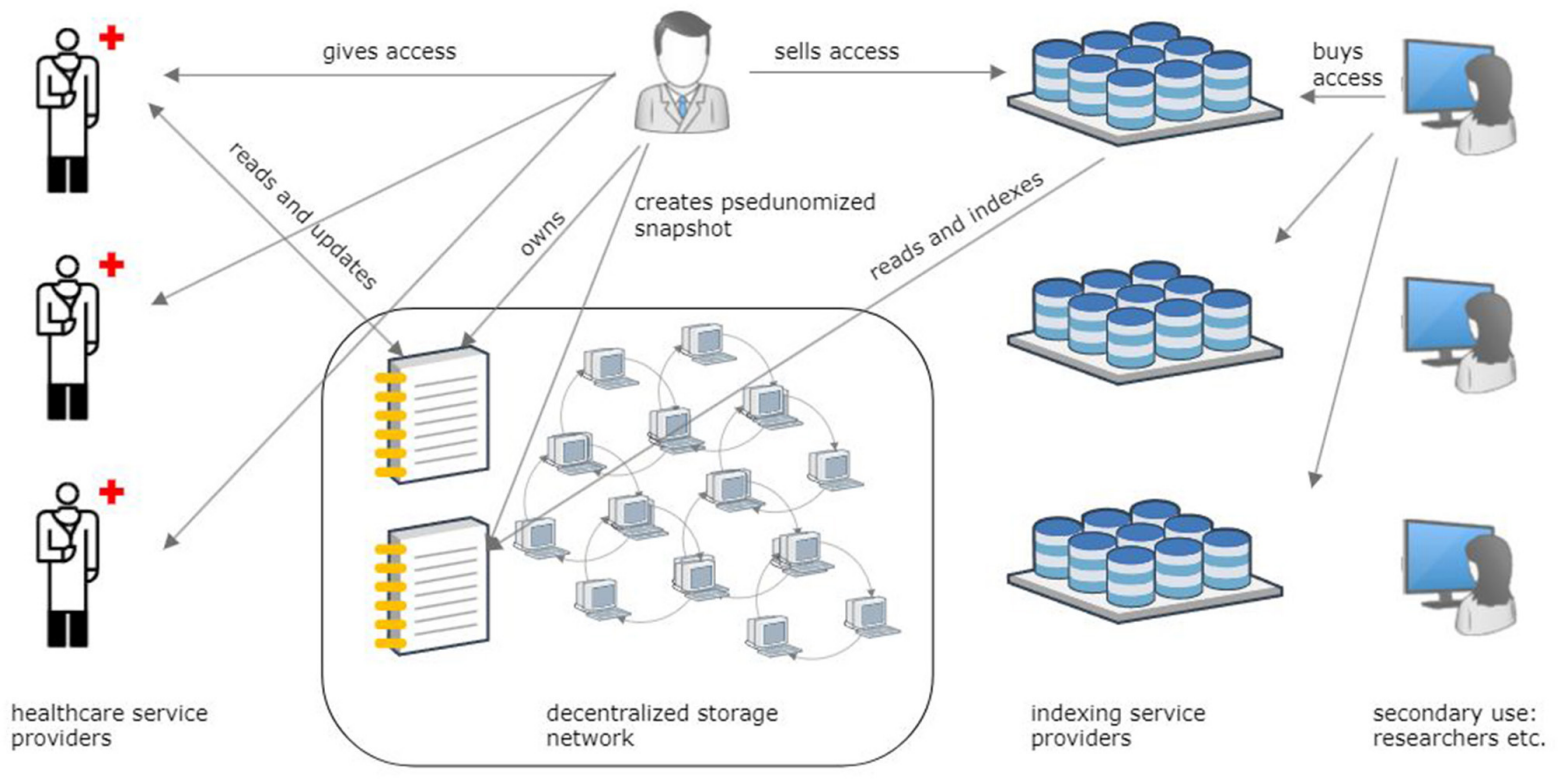
Overview of the reference architecture for storing personal health records in a decentralized content-addressable storage network and sharing health data for primary and secondary purposes.

We propose and evaluate a reference architecture for a Personal Health Data Space based on DCAS networks (Figure 1). The focus of this paper is twofold. The first objective is to outline the typical use cases of health data for primary and secondary use based on existing health information systems (AS-IS) and to explain these systems' inability to resolve the three dilemmas. The second objective is to envisage an innovative DCAS network-based reference architecture for health data management (TO-BE), analyze its properties from the accessibility, comprehensiveness, and ownership dilemma perspectives, and evaluate security, data protection, scalability, and other aspects of the proposed solution under the typical primary and secondary use case scenarios.

The EHIS covers all Estonian residents and is one of the best digital health platforms (35). The Estonian model, operational since 2008 (36), provides valuable experiences that can be extrapolated for broader application. Our research utilizes four common health data use cases from the EHIS. Through this exploration, we shed light on issues and challenges associated with preserving health data within analogous unified national health data repositories. Our analysis underscores the need for cohesive solutions at the EU level, facilitating the seamless exchange of health data across institutional and national borders. Our discussion operates within the framework outlined by the GDPR (29) and the EHDS (37). This involves managing citizens' health data responsibly, ensuring data privacy, and enabling the reusability of health data for societal benefit. We posit that such a system establishes the groundwork for a fair data economy (38), wherein enterprises, especially small and medium-sized enterprises (SMEs), can engage in an innovative business landscape for intelligent health solutions. Simultaneously, citizens gain control over the utilization of their health data and actively participate in a just compensation mechanism, ensuring the equitable distribution of profits generated from innovative solutions based on their data.

**Figure 2:**
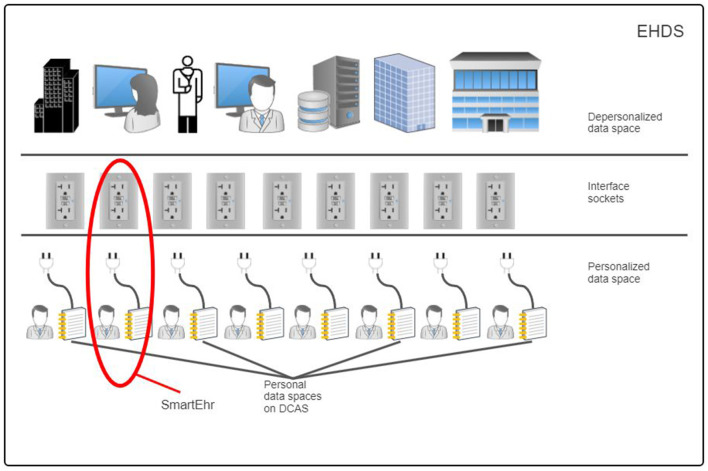
The personal data space in the decentralized content-addressable storage network is valuable for existing hospital, regional, and national health information systems for secure and sustainable retention of personal health data and to support semantic interoperability in data exchange.

The suggested reference architecture is in harmony with the fundamental principles of the European Health Data Space (EHDS) regulation proposal (Figure 2), significantly improving health data management by ensuring security, cost-efficiency, and sustainability. This architecture guarantees individuals' ownership and complete control over their health information while enabling semantic interoperability with existing hospital, regional, and national systems and respecting privacy and data protection laws. Through this solution, people have the opportunity to amalgamate their health information from diverse sources—various healthcare institutions, mobile applications, medical devices for home use, and personal health notes—into a single, integrated Personal Health Record [PHR; (39)]. This all-encompassing PHR can be shared with healthcare professionals, independent of the healthcare provider's location or the type of information system in use. Moreover, this solution empowers individuals to share their de-identified (anonymous or pseudonymous) health data for secondary use for the benefit of society according to explicit legal consent.

The rest of the paper is organized as follows: Section 2 delves into four health data use case scenarios based on the EHIS—primary medical use, medical emergency use, secondary use, and personal use. These EHIS scenarios are then examined through accessibility, comprehensiveness, and ownership to advocate the need for health data management based on DCAS network technology. Section 3 proposes the reference architecture to resolve health data accessibility, comprehensiveness, and ownership dilemmas through preserving semantically interoperable PHRs in DCAS networks. Section 4 evaluates and assesses the critical attributes of the proposed architecture. Section 5 compares the solutions with similar existing ones and examines their integration with existing health information systems and alignment with the EHDS initiative (37).

## 2 Methods

**Figure 3:**
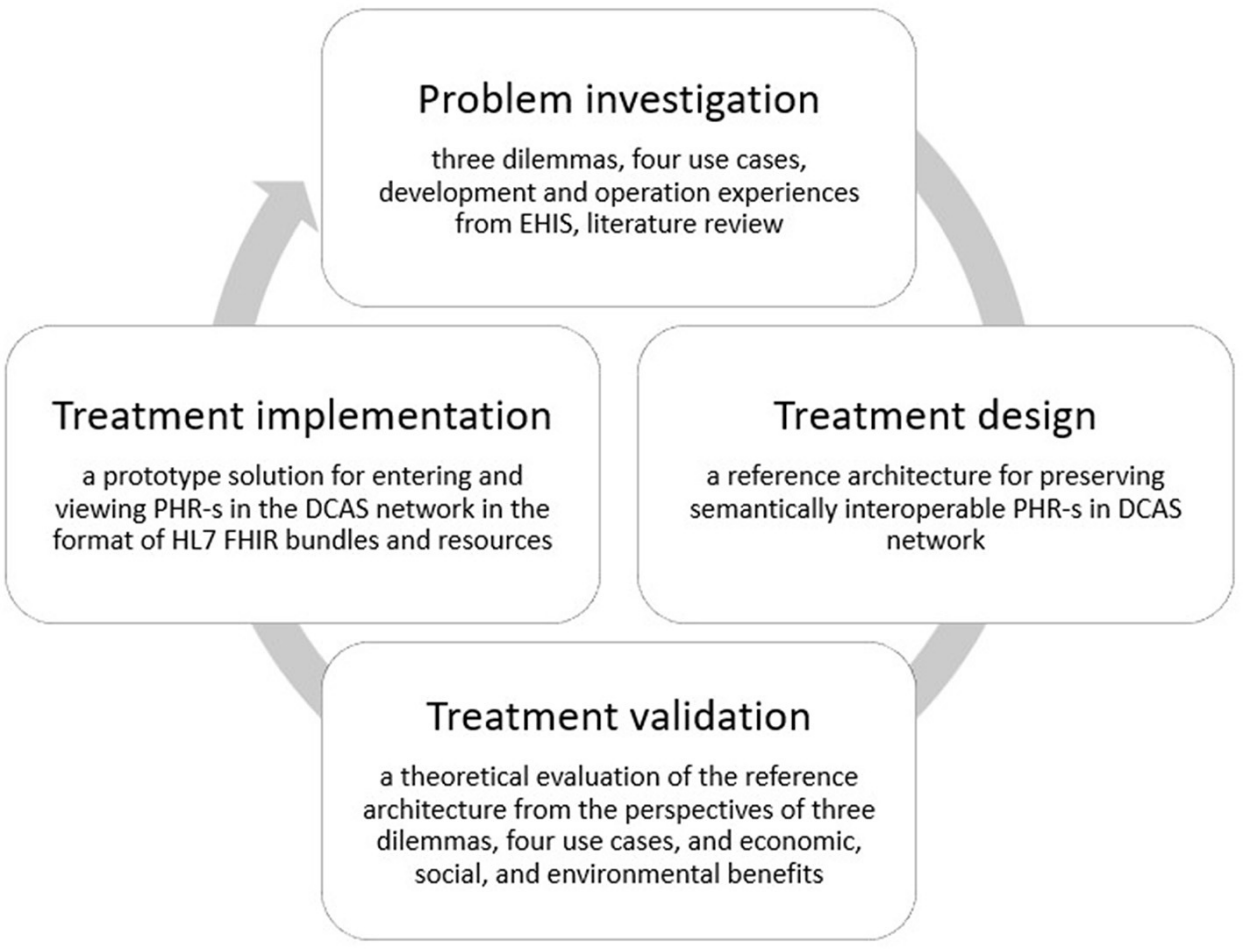
The design science methodology used in the development of the proposed reference architecture.

We adhere to the Design Science (DS) methodology (40), Figure 3, encompassing three steps: (1) investigating a problem, (2) designing a solution (treatment design), and (3) evaluating the solution's effectiveness in addressing the problem (treatment validation). While treatment implementation is not part of DS but is part of the engineering cycle, the figure shows treatment implementation to demonstrate the place and role of the prototype solution in our study.

We articulate the problem through three dilemmas: data accessibility, data comprehensiveness, and data ownership (Section 1). Our analysis is based on a literature review and experiences in EHIS operation and handling. We first describe four use cases (this section, Section 2) based on EHIS operation and explain, based on these use cases, why even national systems like the EHIS fail to address the three dilemmas. As a solution (Section 3), we propose keeping the master copy of the PHR of each person's health record on the DCAS network under the complete control and ownership of the data subject. We will then show (Section 4) how the proposed solution will effectively address the three formulated dilemmas when utilizing the same four use case scenarios and explain how the proposed system supports seamless and coherent interoperability with the existing hospital, regional, and national information systems and data registers.

**Figure 4:**
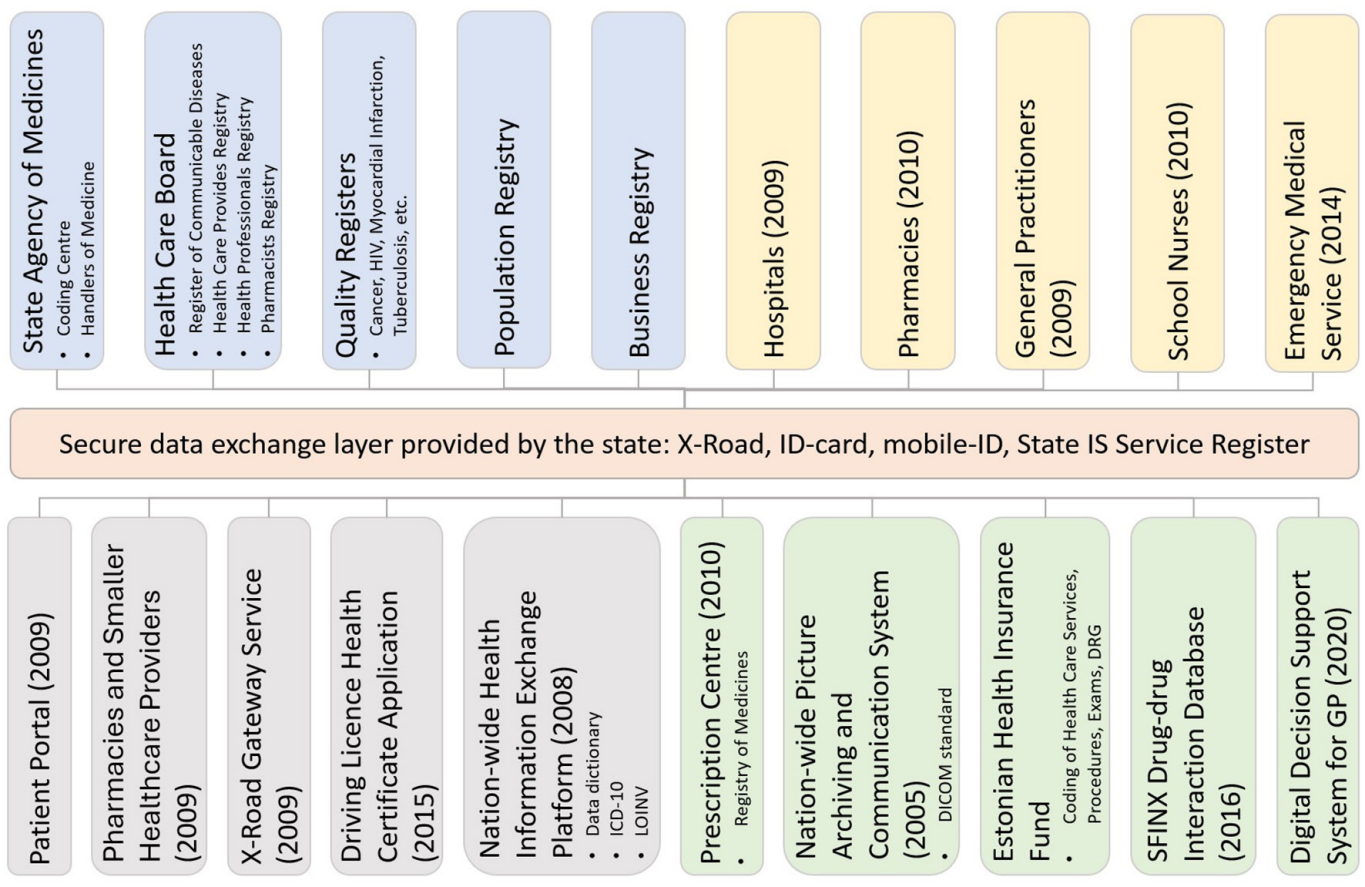
The Estonian National Health Information System architecture. The coloring schema is as follows: orange—central government infrastructure services; blue—national registers; yellow—integrated health service providers; gray—services that either use or provide services to EHIS; green—the central services of EHIS. The year shown in brackets indicates the year of deployment.

The Estonian Health Information System (EHIS, Figure 4) is a central national DHP through which health service providers, such as doctors, nurses, midwives, physiotherapists, and other healthcare professionals, can exchange data and see health data entered by other healthcare professionals about a patient. The EHIS consists of (1) central national databases, e.g., EHR, Prescription Centre, and Picture Archiving and Communication System (PACS); (2) digital health services built on the existing e-government infrastructure, e.g., digital prescription, e-referral, e-consultation, and e-ambulance; and (3) digital decision support systems and cross-sectoral services exploiting nationwide databases, e.g., drug-drug interaction database, clinical decision support system (DSS) for primary care, patient summary. The EHIS provides secure, robust, and reliable internet-based data exchange services for healthcare providers and natural persons. Healthcare service providers must, according to law, transfer specific, defined, structured, and standardized data to the EHIS. Data exchange between healthcare providers and the EHIS is ensured by implementing international standards, such as HL7 CDA and LOINC. Persons can access the EHIS through the Health Portal (41) (available in Estonian, English, and Russian).

In the case of the EHIS and the Health Portal, it is important to note their inseparable connection to other e-government services and tools in Estonia. The EHIS relies on a comprehensive information technology base infrastructure developed at the national level and is a central electronic database where residents' health history is recorded from birth to death. Technically, the health information system has been implemented on top of the state infrastructure solutions [ID card and mobile ID, (42), X-tee, (43), etc.] that most Estonians use extensively. The system is successfully connected to other information technology solutions offered to Estonian citizens, making it convenient for all users. According to the United Nation's E-Government survey, Estonia ranks very high in the E-Government Development Index (44), which might explain peoples' positive attitude toward the Health Portal.

### 2.1 Medical primary use case

A healthcare institution's internal and external information systems and databases are used in the daily work of doctors, nurses, and other healthcare professionals. Electronic Medical Records (EMR) and other Clinical Information Systems are the central in-house clinical information systems. For patient management, healthcare professionals primarily use the EMR. In Estonia, most clinical processes in healthcare institutions have been digitized. Still, paper-based documents have not disappeared entirely, e.g., intensive care spreadsheets, hospital internal orders, nurses' notes. The integrated EMR seamlessly communicates with external information systems if the person has been treated in another healthcare institution in Estonia, a healthcare worker wants to see previous data, or a doctor needs some central services, such as clinical DSS or e-consultation. If the person has been imaged or lab tests have been performed in other institutions, the EMR can query and retrieve relevant images from the nationwide PACS or receive lab test results from another EMR or EHR system. One very convenient service is a digital prescription: the doctor issues a prescription in the EMR, which uploads the digital prescription to the central prescription center after making several queries from national databases, e.g., to find out the reimbursement rate given to the specific patient. Since all digital documents used in healthcare in Estonia are linked to a person's unique personal code, the patient can go to any pharmacy and show their ID code. The pharmacist will immediately see all prescriptions issued for the patient and dispense the appropriate medicine to the patient. E-referral, e-consultation, and other digital health services follow similar principles. Documents completed in the healthcare institution, examination reports, or test results are converted by the EMR into a standard data exchange form and sent to the EHIS, where they are parsed and kept in different repositories. This allows clinical systems to compose either a time series based on data collected in the EHIS from various healthcare institutions, e.g., the dynamics of lab test results over time, or a standard Patient Summary (45). The benefits of a centrally developed, integrated, secure, internet-based, standard-following DHP such as the EHIS are related to data availability, sharing, and security. The medical professional gets a complete overview of the patient's contacts in the healthcare system and their content.

### 2.2 Medical emergency use case

The work of ambulance and emergency medicine departments has been digitalized in Estonia. Paramedics use tablet devices with specially designed e-ambulance software to enter data. E-ambulance and emergency medicine software are integrated with the EHIS (Figure 4). This way, the paramedic can see the patient's previous health data at the scene. The data available to paramedics is not limited to the text or diagnoses; previous medical imaging reports and electrocardiograms (ECG) can also be viewed. The ambulance can use the software to transmit critical information about the patient to the hospital before the patient arrives.

### 2.3 Secondary use case

Unfortunately, health data secondary use for public health, clinical research, medical claims management, or the pharmaceutical industry does not yet benefit significantly from the EHIS. In the EHIS, secure data exchange between various clinical parties is resolved well, but ensuring data quality still has issues and challenges. Although various international classifications and terminologies are in use, their use is insufficient, and medical records still contain a lot of free text. This forces the National Institute for Health Development (NIHD), responsible for public health in Estonia, to collect data separately through the information systems they developed. This causes data duplication and discrepancies.

Firstly, the NIHD collects most of its data through its internet portal, a legally mandated data entry system for healthcare providers to report to the NIHD. This portal, in combination with other government data collection systems, e.g., the EHIS, can be seen as a redundant system and duplicate data entry. The data NIHD collects is often available in other systems, but due to the gaps in data quality and interoperability, it cannot be automatically transferred to the NIHD databases. Secondly, data entered directly into NIHD systems and cleansed for better quality is not shared back in an interoperable way to clinical/administrative healthcare systems. This limits the value of the NIHD's data and analytics, as it cannot contribute to the general quality enhancement of clinical and administrative decision-making processes in hospitals.

The same trend of data being collected in separate information systems can be observed in the case of randomized clinical trials conducted by pharmaceutical companies. However, new registries, such as the Breast Cancer Screening Registry, have been started, which query data directly from the EHIS. Still, systemic weaknesses in cross-sectoral and cross-institutional regulation, coordination, and clinical data standardization limit the secondary use of health data. This creates a need for manual data processing and culminates in inefficient information handling and systems development.

Hospitals often use several software applications for administrative data when automated integration with medical systems is not in place. Frequently, manual data entry is needed for reporting and statistics. In most hospitals, the raw data is electronic but manually transferred for reporting and statistics. Additionally, regulations on the health information system, prescription system, reimbursement system, public health reporting system, or vertical registries (cancer, HIV, tuberculosis, myocardial infarction, etc.) are not always harmonized, or the clinical information classes are defined too generally to be usable practically. Therefore, each responsible agency, specialty, or sector develops its terminologies and data structures independently. This leads to point-to-point solutions, lessens system interoperability, and ultimately increases manual data processing and complicates software development.

### 2.4 Personal primary use case

**Figure 5:**
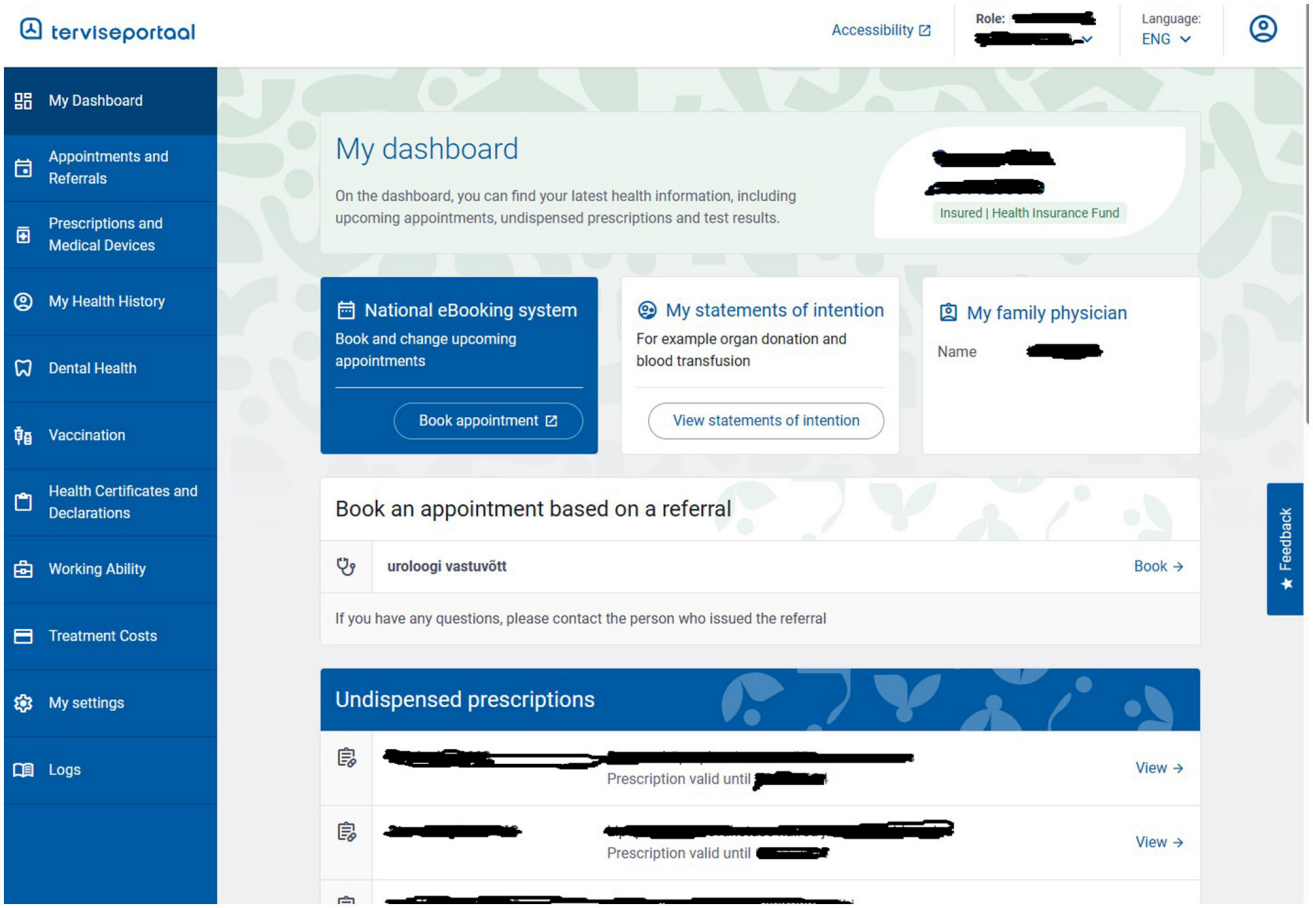
A screenshot from the Health Portal of the Estonian National Health Information System.

In the Health Portal (Figure 5) of the EHIS, a person can see their health and medical data and may perform several activities. This data has been collected according to how the person's treating physician or healthcare institution sent them to the EHIS in a standardized way. A person can submit declarations of intent, appoint a representative, perform actions on their behalf and on behalf of the person represented, and view the medical invoices submitted by healthcare institutions to the Estonian Health Insurance Fund about their medical treatment. All prescriptions in Estonia are in digital form, and a person can see the issued prescriptions and their status in the portal.

All residents can access their data to determine their consent for specific health data sections. This means the patient can restrict access to certain documents, medical records, and all personal data in health information systems. Access restrictions can be imposed on one individual document or all information contained in the EHIS. From the point of view of data security and privacy, it is essential to note that a person can monitor all activity logs in the Health Portal, i.e., see which medical professional has requested their data and when and what document was viewed.

### 2.5 EHIS from the perspectives of the three dilemmas

The Estonian National Health Information System (EHIS) is a pioneer in digitizing healthcare on the national level. However, the system faces significant challenges related to the dilemmas of accessibility, comprehensiveness, and ownership.

1. *Accessibility*: The EHIS fails to resolve the accessibility dilemma as it lacks features for secondary data usage, as previously mentioned. Consequently, the initial aspect of the dilemma, necessitating data access, remains unresolved. Moreover, the EHIS falls short in ensuring comprehensive protection of personal data, as its measures aimed at limiting access are reactive rather than preventive. While data owners can detect unauthorized access, they cannot preemptively exclude it.
2. *Comprehensiveness*: The EHIS fails to resolve the dilemma of comprehensiveness primarily because, at the global level, it operates as an isolated data silo. Moreover, even at the local scale, the EHIS does not provide a holistic perspective of an individual's health profile. Research suggests that patient data stored within healthcare facilities tends to be more accurate and thorough than EHIS data (46). Additionally, the exclusion of patient-generated data, such as lifestyle information and data collected from wearable devices, further restricts the system's capacity to offer the complete picture. Consequently, the EHIS merely presents a simplified and partial representation of the data, contradicting its initial aspirations for comprehensiveness.
3. *Ownership*: The EHIS fails to resolve the ownership dilemma, as the institution managing the data retains physical control. While the data subject possesses certain rights, such as the ability to restrict access to specific data and monitor the audit trail of data usage, the managing institution remains the de facto owner of the data. This scenario resembles feudal land ownership relations, where the land belongs to the landlord, and the peasant has limited rights to utilize part of it for personal use.

To surmount these challenges, a different approach is needed—one that embraces decentralized technology to enhance system agility, incorporates patient-generated and -entered health data to ensure data comprehensiveness, and empowers patients with preemptive and complete control over their health information. Such a system would facilitate seamless cross-border health data exchange, support the integration of innovative health technologies, and streamline consent management for secondary data use.

## 3 A reference architecture for personal health records

### 3.1 An overview of the architecture and fundamentals

The proposed architectural solution to solve the three dilemmas is based on the novel decentralized content-addressable storage (DCAS) network technology (Figure 1). We first analyze data management risks to grasp the principles by which DCAS networks operate.

By aggregating all health data in one place and keeping it in a hospital, regional, or national health information system, the risk of data management increases due to a single point of failure, attractiveness to attackers, the complexity of security management, difficulties in access management, and the complexity of regulatory requirements. The opposite also applies—splitting a large dataset into smaller components reduces the risk of managing each component and the whole. Continuing this iterative data volume-reducing process leads to a scenario where the risk linked to an individual tiny data fragment approaches zero, and the implementation of intricate and costly security measures becomes superfluous.

DCAS networks operate on a similar principle. They are peer-to-peer networks wherein nodes run open-source software designed to store an enormous quantity of tiny data fragments. When some data, such as a file or a document, is to be stored in such a network, the data is first split into data fragments of a few kilobytes each. These fragments are then distributed across various nodes according to the network protocol. Each fragment represents an insignificant fraction of the complete dataset, making it feasible to distribute them between nodes without jeopardizing the privacy of the entire dataset. As the anonymity of DCAS network nodes is part of the DCAS protocols, the trustworthiness of the node operators is not imperative for secure data storage within the network, as individual data fragments are not informative. In addition, no node knows to which dataset the fragment belongs, the location of nodes, or the nodes where the remaining data fractions are stored.

**Figure 6:**
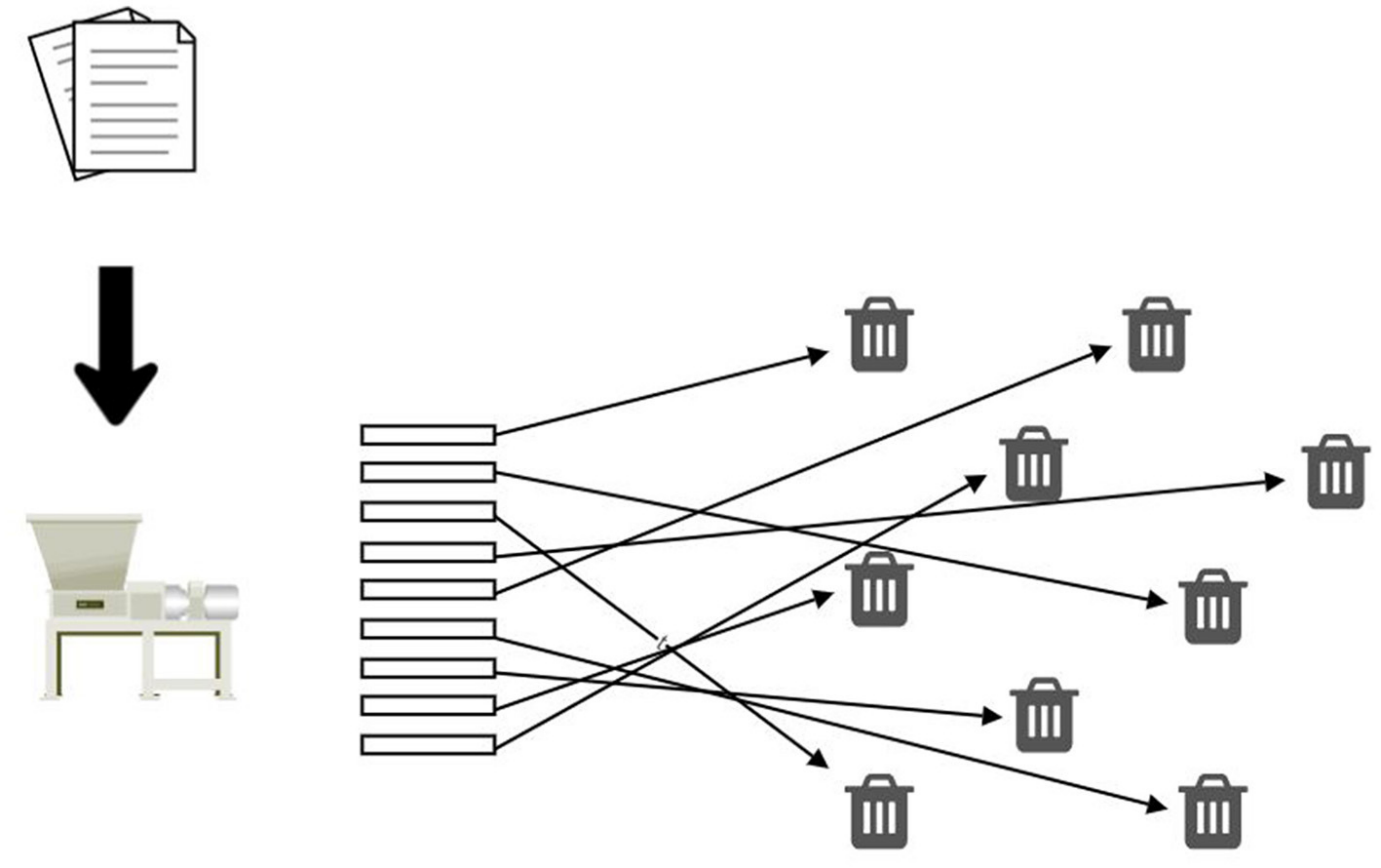
The decentralized content-addressable storage as an electronic paper shredder.

Conceptually, a DCAS network resembles a paper shredder (Figure 6), cutting a classified document into tiny strips, none of which divulge the document's contents. Unlike a physical shredder, a software-based implementation can reconstruct the original document from its shredded components. This reversal process merely necessitates knowledge of the root hash of the original document, which a data owner must only keep to themselves. Here and in the future, a data owner means a person who keeps their data on a DCAS network and, if necessary, shares that data for primary or secondary use.

In the following, we provide concise overviews of the fundamental characteristics of a DCAS network. While Ethereum Swarm (47) has inspired these descriptions, they are formulated broadly enough to apply to any implementation of a DCAS network.

***Content addressing*. **In contemporary internet architecture, location-based addressing is widely employed. The typical structure of a web URL consists of several components: the server name, which is substituted by the IP address during the name resolution process, the name of the sought-after resource, and the path to the directory where the desired resource is situated. This method of addressing is called location-based addressing, as the resource's address signifies its physical location.

In contrast, content-based addressing is not based on the location of a resource but highlights its content (48). Content-based addressing, in many respects, is more intuitive than location-based addressing. When searching for a specific resource, its content is of primary importance rather than its physical location. This can be observed in everyday activities like shopping. In a store, individuals are not concerned with the product's precise shelf but are interested in milk or bread, irrespective of their spatial arrangement.

DCAS networks use content-based addressing. Each network node has an overlay address, a randomly generated integer. To avoid duplication of overlay addresses, large, 256-bit integers are used. The Kademlia distance (49) between two network nodes is an integer obtained by the exclusive logical addition (XOR) of overlay addresses of nodes. For instance, the Kademlia distance between overlay addresses 5 (0101) and 6 (0110) is 3 (0011). The Kademlia distance has all the fundamental characteristics of distance, including non-negativity, symmetry, the zero value of a node's distance from itself, and triangle inequality. In the DCAS network, each shard of information is stored on the node whose Kademlia distance is closest to the shard's hash value. The hashes of the shards are arranged into a Merkle tree (50), which is stored in the DCAS network following the same information-splitting protocol. The hash value of the Merkle tree's root serves as the address of stored data.

When retrieving data from the DCAS network, the process is reversed. Specifically, the network protocol implemented in the node software locates the node closest to the given hash value and finds its underlying address (IP address in the context of the internet). Subsequently, a request is sent to this identified node to access the desired data. Content addressability serves as a supplementary measure to ensure data integrity. This is achieved by enabling the consumer to verify the content of the downloaded data by calculating its hash value and comparing it to its address, thus confirming that the data has not been altered.

***Decentralization***. Firstly, let's delve into some terminological considerations. The term “decentralized” is frequently employed to convey the idea of a system comprising numerous smaller, independent entities. To illustrate, a “decentralized data network” is commonly understood as a federation of diverse data sources, each independently comprehensive within localized boundaries (51). This implies that information about a specific subject is internally cohesive within these local confines. While these data sources may lack global completeness by not encompassing all available information about a particular topic, they wield control over the data within their purview.

However, this paper adopts a more stringent definition for “decentralized”, signifying a system where data lacks completeness even locally, information stored on individual nodes is indecipherable, and no governing body exists locally or globally. The absence of a governing body within the DCAS network means that independent node operators individually determine all decisions, including joining or leaving the network. At the same time, the network protocol incentivizes each node to make decisions that contribute to the network's objectives.

***Redundancy***. Network decentralization refers to the absence of a governing authority body within the network (52). Consequently, network nodes can disconnect from the network at any given moment. Upon leaving, these nodes take with them the data shards they have been storing. This presents a significant challenge, as restoring the data that these shards were part of is impossible. Naturally, such a situation is unacceptable, necessitating the implementation of redundancy to prevent data loss.

One potential approach to address redundancy involves storing each piece of data not only on the node closest to it based on Kademlia distance, but also on a set of nodes that belong to a specific neighborhood of responsibility surrounding the closest node (53). Since overlay addresses are randomly assigned to the network nodes, and the Kademlia distance has nothing to do with geographical dimensions, network nodes belonging to this neighborhood are typically dispersed worldwide under the management of different operators. Based on network size and its rate of churn, a sufficiently large radius of the neighborhood can be chosen, ensuring that the loss of a single piece of data resulting from the departure of the node storing it is close to zero (54).

The outlined redundancy method represents just one approach to guarantee data redundancy. Alternatively, more efficient techniques like Erasure Coding (55) may be used. Despite distinct algorithms, the objective remains to ensure data preservation within the network when nodes exit the network.

***Immutability and de-duplication***. Content addressability leads directly to the immutability of the data (56). This is due to using hash values as addresses, where any change in the content of the data results in a change in its address. Consequently, the altered data becomes a new addressable entity for the network, while the previous version remains accessible at the earlier address. Therefore, the DCAS networks inherently retain the version history of any data modifications.

As described, the data is typically fragmented into tiny pieces stored independently as individual entities within the DCAS network. Likely, only a particular portion of these pieces will be modified when changes occur in the data. Those pieces that remain unaltered continue to exist online at their former addresses. Thus, DCAS networks efficiently maintain the version history of the dataset, ensuring that only one copy of the data exists within the network, excluding the copies required for redundancy.

***Mutable address space***. Content addressability has numerous advantages (57). As previously mentioned, content addressability results in data immutability, as any modification to the data corresponds to a change in its address. However, there are cases where it becomes essential to store mutable data at a designated address. To accommodate this need, each user in a DCAS network is allocated a personal mutable address space. This dedicated space allows users to manage and modify data within specific addresses without conflicting with the immutability constraints associated with content addressability.

***Incentives***. Decentralized networks' successful emergence and sustainability rely on establishing a precise and robust incentivization mechanism (58). This mechanism must adequately motivate network operators to bear the costs associated with providing services and is typically facilitated through compensation from the users of the network services. However, the absence of a central governing authority poses a challenge in orchestrating this compensation process.

Adopting a compensation mechanism built on blockchain and smart contracts is imperative to achieve incentives in complete network decentralization (59). Within such systems, it is feasible to use crypto tokens for payment. Ethereum Swarm, which operates on the BZZ crypto tokens (60), is an example of a decentralized compensation mechanism implementation. Alternative compensation mechanisms have also been implemented. However, any method reliant on traditional fiat currency necessitates the involvement of an intermediary body, compromising the network's decentralization.

### 3.2 Core application

**Figure 7:**
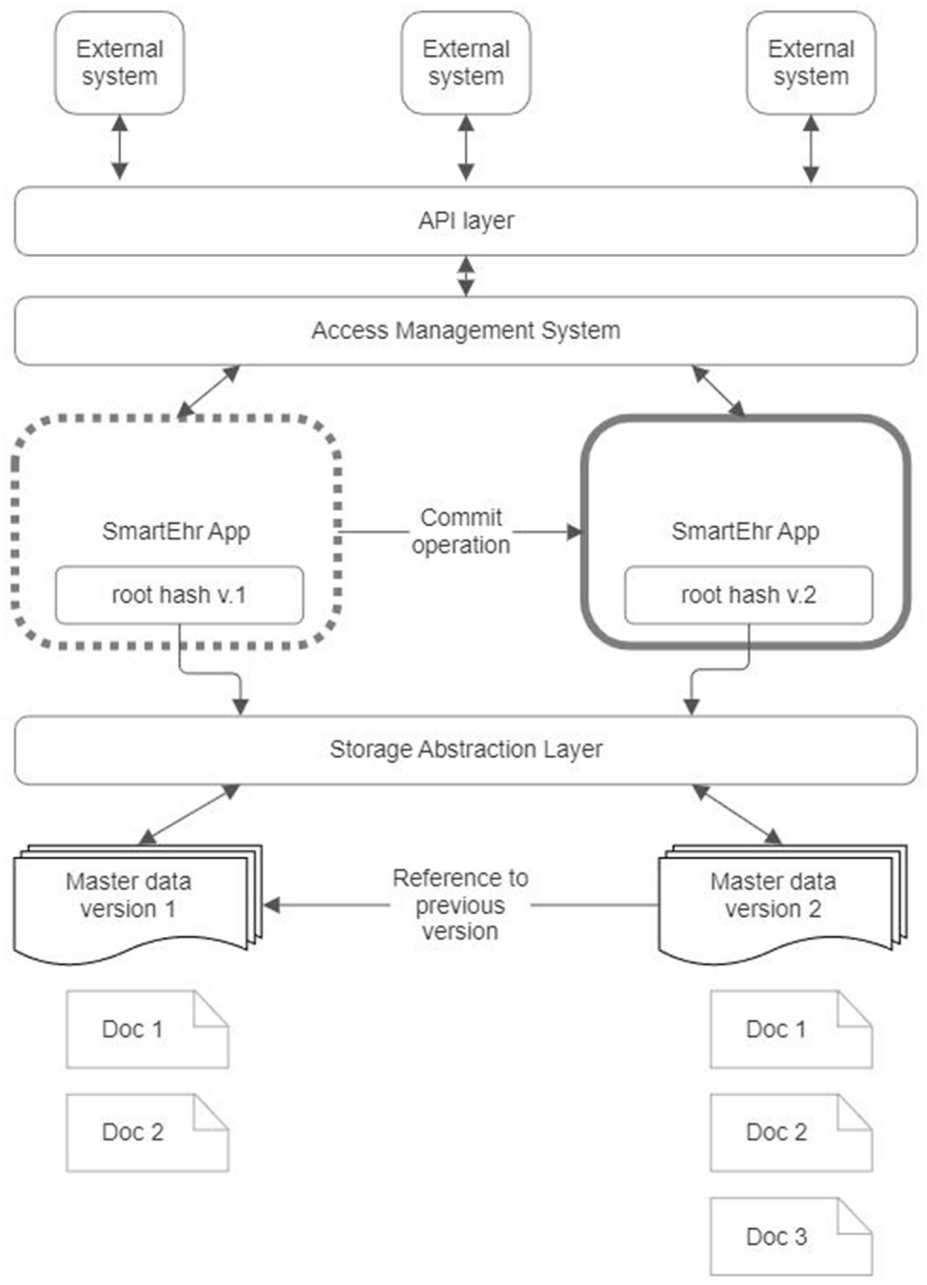
Core application architecture of the proposed reference architecture for personal health record.

The core application (Figure 7) is open-source software that operates on the user's device, serving as a personal portal to health data. This application primarily aims to present a person's health data stored online in a DCAS network in a user-friendly manner. Additionally, it enables persons to perform various tasks such as annotating, searching, filtering, and sorting health information. Furthermore, it establishes data communication with the DCAS network using an abstraction layer, which ensures independence from the implementation of a specific DCAS network. Moreover, the core application employs software layers to incorporate protocols and standards commonly used in healthcare to facilitate interoperability. The core application's functionality can be expanded by integrating separate downloadable modules.

The following subsections present a detailed description for each component of the core application.

#### 3.2.1 Root hash management

The root hash granting access to the data should be known to the data owner exclusively. This hash value plays a crucial role in granting access to the data; therefore, the data owner must thoroughly protect it. In the unfortunate event of losing the hash value, retrieving access to the data becomes impossible. Consequently, the method employed for storing the hash value must incorporate safeguards to prevent both unauthorized access and accidental loss; therefore, safeguarding and securing this hash value is a primary responsibility of the core application.

Whenever a modification is made to the data, the hash value is updated to reflect the changes. The new hash value permits access to the modified data, while the previous hash value represents the prior version of the data. Preserving the entire version history of the data within the core application may not be feasible due to practical limitations. A possible approach is to include the address to the previous data version within the data itself. This enables the core application to retain the whole history of data amendments.

In addition, it is essential to consider the possibility of the stored hash value being unavailable due to, e.g., the data owner's device being lost. In this case, storage of a constantly changing hash value in a recoverable manner poses a significant challenge. A plausible alternative involves storing the changing root hash within the DCAS network. This is where the personal mutable address space of the DCAS network proves valuable. By storing the encrypted hash value within the user's private mutable address space, the core application simplifies its task to retaining the constant address where the current root hash resides.

**Figure 8:**
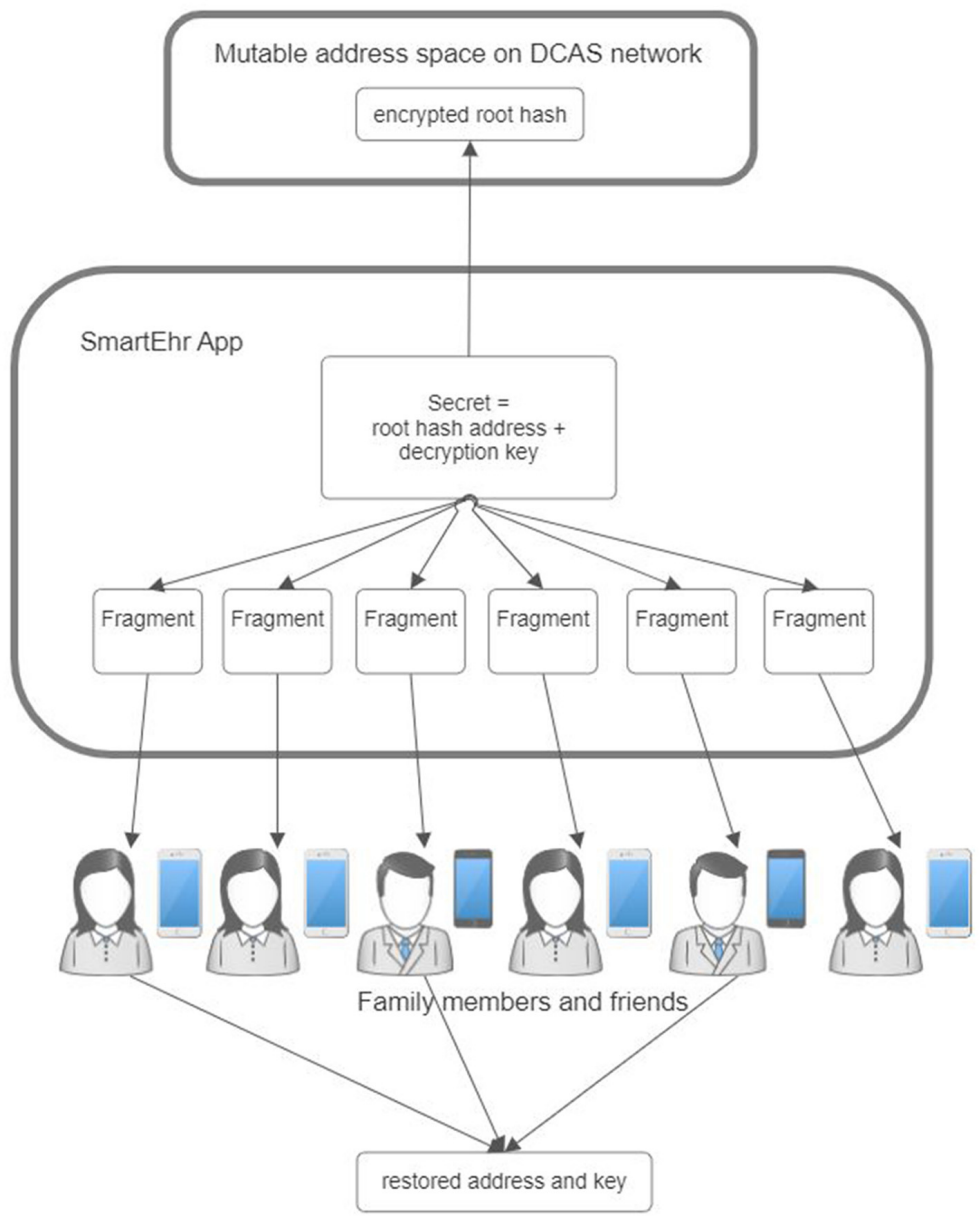
A root hash management in the proposed reference architecture for personal health record.

This constant value facilitates the implementation of secret sharing algorithms, like Shamir's Secret Sharing (61), to effectively mitigate the risk of data loss. This secret-sharing framework mathematically divides the constant address where the current root hash resides into multiple shares (Figure 8). Each share is then stored separately in the main applications of the data owner's closest relatives so that only one share is retained by one relative. This secret-sharing mechanism ensures that the address can be recovered by gathering a sufficient number of shares that meet or exceed the predetermined threshold. Conversely, it is impossible to reveal the secret address if the number of shares is below that threshold.

The solution above also provides a means to safeguard data against unauthorized modification. In this approach, recording the hash value of the modified data is exclusively permitted in the owner's mutable address space. Consequently, any alteration to the data necessitates the owner's approval by storing the revised hash of modified data. This confirmation process can be likened to the commit operation commonly employed in databases. Without such confirmation, any changes to the data are lost.

#### 3.2.2 Storage abstraction layer

The Storage Abstraction Layer (SAL) is an intermediary component, facilitating communication between the core application and the DCAS network. This intermediary layer ensures the core application's independence from the specific implementation details of the storage network. It aligns with the principle of dependency inversion commonly employed in software development. Incorporating an intermediate layer such as SAL, the core application can remain unaffected if replacement of the layer becomes necessary. The core application solely requires functionality related to the reading and writing of data, while SAL effectively manages all other intricacies.

#### 3.2.3 Content handlers

Numerous standards exist to represent health data, including various HL7 standards and versions, OpenEHR (62), ISO 13606 (63), and ContSys (64). It is desirable for the core application to not be restricted solely to clinical data but to offer the capability of managing diverse information about an individual's health and general lifestyle. As this data can be generated by various devices from different manufacturers, they might exhibit disparate formats and employ distinct data models. Content handlers in the core application are to handle this multitude of data models effectively. These autonomous software modules adhere to the dependency inversion principle, akin to the Storage Abstraction Layer. Incorporating these content handlers into the core application does not necessitate any modifications to the core application itself. The data should be presented online in a self-descriptive manner, enabling the bootloader to select the appropriate content handler for processing.

#### 3.2.4 Interoperability layers

The purpose of the interoperability layers is to facilitate the integration of the core application with external information systems. A key objective of these layers is to enable healthcare providers to access patient data in the context of primary and secondary use. As previously mentioned, one data-sharing approach involves disclosing the data address (its root hash). Nevertheless, a preferable alternative is to grant data access via an application programming interface (API), such as FHIR, which preserves the confidentiality of the root hash. It is reasonable to use federated [on-the-fly adaptation according to the third-party data exchange protocol; (65–67)] rather than integrated (based on a standard data format) or unified (based on a common standard) interoperability (68) to achieve flexible and adaptable interoperability across hospital, regional, and national health information systems.

#### 3.2.5 Extension modules

Extension modules are plugins that serve the purpose of augmenting the existing capabilities of the core application. These supplementary features encompass various enhancements, such as integrating diverse wearable devices into the core application and incorporating various algorithms enabling individuals to supervise and assess their health-related behaviors. It is vital to note that these extension modules obtain access to individuals' PHR through the core application, while concurrently enabling other system components to harness the data they generate.

## 4 Evaluation of the proposed architecture

### 4.1 Practical experiments

Practical experiments were conducted to validate the viability of the proposed reference architecture. Due to the sensitivity surrounding medical data and the constraints imposed by legal regulations, obtaining medical data for testing poses significant challenges. Instead, we used the Synthea package (69) to generate synthetic health data. Synthea is an open-source data generator renowned for producing realistic medical history data for synthetic patients, encompassing various healthcare scenarios. It allows for the creation of datasets of any desired magnitude. For our experiment, a dataset comprising 1,000 synthetic persons was generated.

As Synthea generates data in the format of FHIR bundle resources, we selected this data format for our experiment. However, it's important to note that our choice of FHIR format does not necessarily imply its superiority in DCAS networks. Ultimately, Resource Description Framework (RDF) and personal knowledge graphs offer more flexible solutions. Since FHIR is also concerned with developing RDF (70) and other concentrated and thin data formats [e.g., FHIR Shorthand (71)], we are likely not far from the desired and practical results to support federated semantic interoperability with a third-party hospital, regional and national healthcare systems, and innovative welfare applications.

We opted for Ethereum Swarm (47) as our DCAS network for several compelling reasons:

1. Full decentralization: Ethereum Swarm operates without a central authority, ensuring a decentralized ecosystem.
2. Robust incentivization: The network boasts a robust mechanism encouraging participation and contribution.
3. Ideal for small data storage: Ethereum Swarm is well-suited for efficiently storing small data fragments, such as FHIR resources.
4. Open-source nature: Ethereum Swarm is open-source and fosters transparency and collaborative development.

The Swarm network comprises independent nodes running the Bee software (72), compatible with both Linux and Windows systems. For our setup, we have chosen Ubuntu Linux as our operating environment. Despite its modest resource requirements, Bee performs optimally with an SSD hard drive and a fast network connection, handling network traffic efficiently.

**Figure 9:**
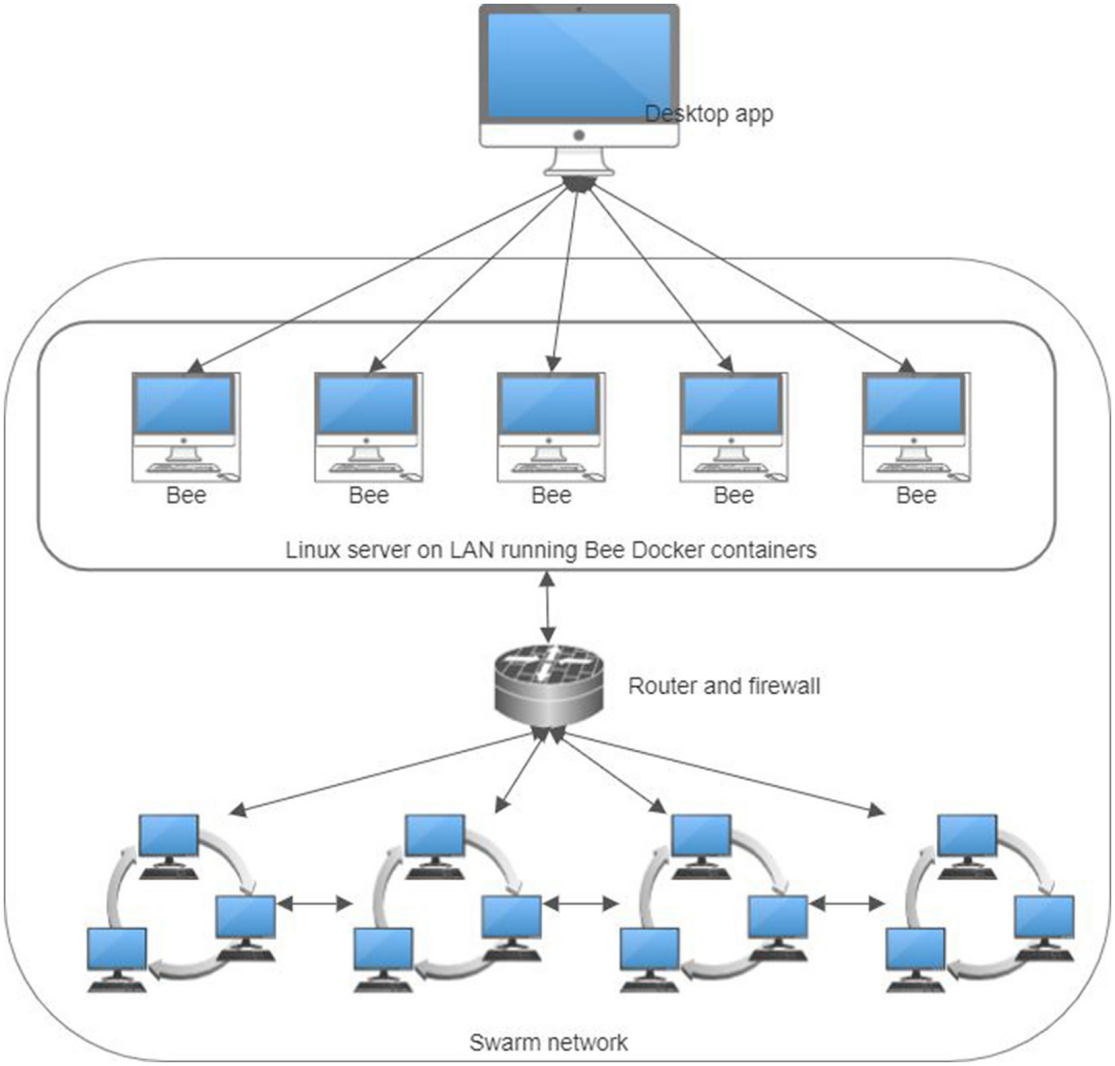
The infrastructure of the practical experiments for storing personal health records in a decentralized content-addressable storage network.

**Figure 10:**
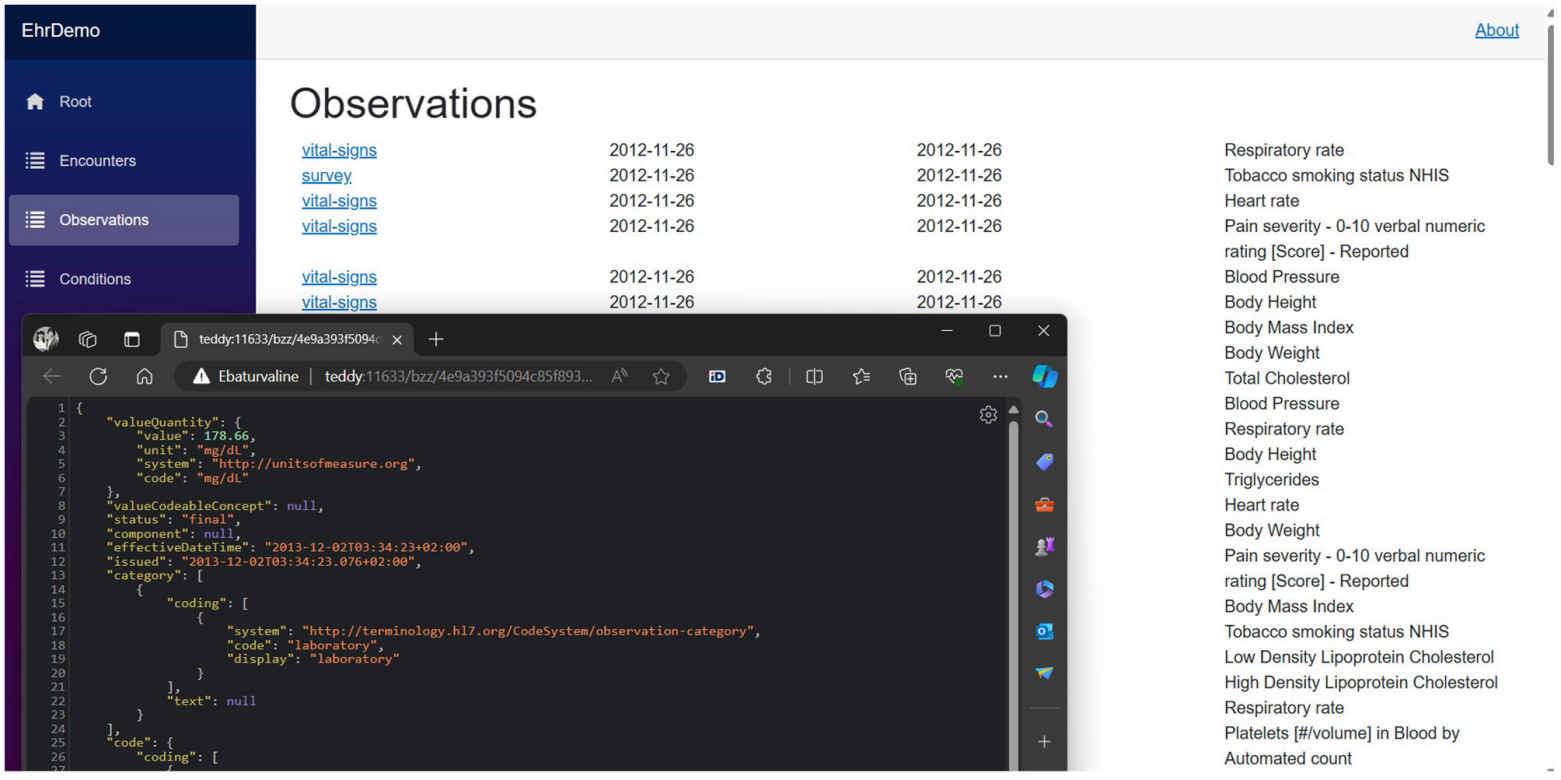
A screenshot of the proof-of-concept app shows a list of FHIR observation resources, with one open in the browser.

The software development environment for this project was Microsoft Visual Studio 2022. The FHIR bundles generated were dissected into individual resources and stored in an SQL Server database to facilitate ease of manipulation. Subsequently, each resource was uploaded to the Swarm network as a distinct entity, uniquely addressed with a hash key. A patient's resource index was stored separately as an FHIR bundle resource, incorporating multiple FHIR Reference resources. The .NET task-based asynchronous pattern (TAP) enhanced query efficiency. A dedicated program in C# was designed to upload the generated FHIR resources. This involved strategically alternating queries between five Bee Docker container nodes and executing 40 simultaneous POST requests in parallel for each, optimizing the uploading process (Figure 9). Parallel queries were similarly employed for data downloads. Due to Swarm's massively parallel protocol, which sends simultaneous requests to numerous network nodes for data chunks, the overall user experience was comparable to, if not better than, traditional web browsing. A screenshot of the experimental app showing a list of the generated FHIR resources stored on the Ethereum Swarm live network is shown in Figure 10.

### 4.2 Medical primary use case

Relying on utilizing DCAS networks to preserve Personal Health Records, the proposed reference architecture (Figure 1) integrates with existing hospital, regional, and national health information systems seamlessly and in a semantically interoperable manner (Figure 2). This architecture features a person-owned application (Figure 7) that operates on the person's device. This application is responsible for securely storing the root hash of the person's health data and facilitating the reading and writing of data within the DCAS network.

In the primary use scenario, a person (data owner) can share data with a healthcare provider by disclosing the root hash of their data (Figure 1). Once the healthcare service provider completes the necessary edits and saves the additions to PHR, a new data version and the corresponding new hash value are generated. The service provider relays the updated value to the data owner, who securely stores it via their application. The healthcare service provider should not retain the original or the revised root hash.

Alternatively, data sharing can occur without disclosing the root hash. One possible method is utilizing a standardized API, such as HL7 FHIR (73), integrated within the data owner's application. However, in such cases, additional measures must be developed to uphold the integrity and reliability of the shared data (74–76).

In medical data, the integrity of information holds paramount importance. A key strategy to ensure data reliability involves the digital signing of entries by the respective contributors. In this context, the data's trustworthiness hinges on the trustworthiness of data entry. Beyond signing the added or modified part of the data, an additional layer of security can be established if the healthcare provider signs the data they enter and the root hash of the entire dataset as it was presented to the healthcare provider during the medical treatment or service provided.

The data subject can conceal specific portions of their data by restricting access for particular healthcare providers. This concealment involves generating a new version of the data, accompanied by a corresponding alteration in the root hash, as elucidated earlier. Significantly, the de-duplication feature outlined earlier clarifies that creating a partially concealed data set does not involve duplicating the entire dataset. Instead, it only stores the modified data fragments in the DCAS network.

When a service provider adds an entry and signs it, they essentially endorse the data they contributed and the entire dataset as it was presented to them. This ensures a comprehensive and signed record of the data collection, offering a transparent snapshot of the information available to the service provider at the time of data entry.

### 4.3 Medical emergency use case

The proposed architecture offers a simple solution for emergency access to an individual's health data. For this, a distinct data subset must be created encompassing vitally important information, such as data about chronic conditions and ailments, medications, allergies, and other related details. These particular data entities form a specialized subset within the comprehensive health data and are endowed with a unique address within a DCAS network, enabling global accessibility. Individuals should consistently carry the reference to this subset, either in digital format stored on a microchip or physically embodied as a QR code on a wearable tag or implemented through alternative means. In a medical emergency, medical personnel can retrieve the most critical health data of the individual by scanning the aforementioned QR code or reading it from the microchip. This method allows access only to the depersonalized subset of health data encompassing vital information during emergencies, while protecting the identity and other PHR data.

### 4.4 Secondary use case

For secondary use (Figure 1), the Personal Health Record must be de-identified (31) to make anonymized or pseudonymized data versions. This process involves the removal of any information that could lead to the identification of the subject, while preserving the reliability of the data. To achieve this, a third party trusted by all stakeholders plays a crucial role. Whether a national institution or a purpose-built organization, this entity verifies the data subject's identity. Subsequently, it validates and removes all signatures associated with the data and appends its own signature to the dataset. This signature proves the reliability of the de-identified data, now derived from the trustworthiness of the third party that signed the data. Through this multifaceted approach, data de-identification not only preserves data subjects' privacy but also ensures the integrity and credibility of the de-identified dataset.

This de-identified dataset is stored within the DCAS network as a separate entity, assigning a new address (hash) to the data. The person may share (possibly for compensation) this hash with third parties interested in utilizing the data for secondary purposes. In real life, the transfer of data from the person to the end user would probably not take place directly but through a data intermediary who aggregates the data of multiple persons and prepares them as a comprehensive data registry for the end-consumers for data analysis.

### 4.5 Personal primary use case

In the context of the DCAS network architecture, the personal primary use case focuses on empowering individuals with complete control over their health data. By leveraging DCAS technology, individuals can manage, share, and protect their health data more effectively, fostering a more personalized and secure healthcare experience.

The cornerstone of the personal primary use case is the individual's ability to consolidate and control their health data through a unified Personal Health Record (PHR). This PHR aggregates information from various healthcare providers, mobile applications, home medical devices, and personal health notes. As the data owner, the individual retains exclusive access to the root hash, ensuring that they control who can access their data and under what circumstances. This control extends to updating, annotating, and managing their health data directly through a user-friendly core application.

One of the critical features of the proposed architecture is its emphasis on semantic interoperability. The PHR can be shared with healthcare providers across regions and systems, ensuring that the data is meaningful and useful regardless of the recipient's technology. This particularly benefits individuals who travel frequently or receive care from multiple providers. Sharing the root hash or utilizing standardized APIs, individuals can grant healthcare professionals access to their up-to-date health records, facilitating informed and timely medical decisions.

The architecture empowers individuals by enhancing transparency and ownership of their health data. Users can monitor all access to their health records. This transparency builds trust in the system and encourages individuals to engage more actively in their healthcare management. The ownership aspect is particularly transformative as it shifts the control of health data from institutions to individuals, enabling them to decide how their data is used and shared.

In addition to primary use, the architecture supports the secondary use of health data while maintaining privacy. Individuals can anonymize or pseudonymize their data and share it for research or commercial purposes. This contributes to societal health benefits and opens up opportunities for individuals to receive compensation for their data. The trusted third-party intermediary ensures that de-identified data remains credible and secure, facilitating its use in various secondary applications.

Integrating Artificial Intelligence (AI) and Machine Learning (ML) algorithms into the DCAS-based reference architecture adds a significant layer of personalization and precision to healthcare management. These technologies can analyze the comprehensive health data stored in the PHR to generate tailored lifestyle and healthcare recommendations. For instance, AI and ML can propose dietary adjustments, exercise plans, or preventive measures based on the individual's health history, genetic information, and real-time data from wearable devices. However, it is crucial to maintain a clear distinction between the recommendations provided to individuals and those given to healthcare professionals. Suggestions for personal use should focus on lifestyle and preventive care, empowering individuals to make informed decisions about their health. In contrast, recommendations for doctors should assist in clinical decision-making, ensuring they have accurate and relevant information to provide the best possible care. This separation is vital to prevent confusion and ensure that clinical advice remains in the domain of qualified healthcare providers.

### 4.6 Resolving the three dilemmas

The dilemma of accessibility is resolved by partitioning the entire personal health data space (Figure 2) in a DCAS network under the complete control and ownership of a data-owning person into distinct non-intersecting sub-spaces of identifiable and de-identified (anonymized or pseudonymized) health data. Identifiable personal health data stored within the former is exclusively controlled by their data owners (data subjects). As long as the root hash of the data remains secret and known solely to the owner, no other party, except those that the owner has explicitly shared the root hash with, has even a theoretical chance of accessing this data. Conversely, the data owner can generate numerous de-identified health data copies with minimal risk of re-identifying the data owner. These copies can be freely shared for secondary use.

The dilemma of comprehensiveness is resolved by consolidating a person's health data from multiple healthcare institutions, portable health devices, health-related applications, and other sources into a complete Personal Health Record (PHR). Since this comprehensive PHR remains under the exclusive physical control of the owner (data subject), the concentration of data does not increase the data leakage risks, as in the event of a successful attack, only one person's data can leak. A master copy of PHR data is used only in cases of initial use of data by sharing this data only with healthcare professionals from desired healthcare facilities regardless of region or national affiliation.

In addition, the ownership dilemma is resolved by storing personal health data within DCAS networks, where access requires the owner's root hash. The network's decentralization ensures that access is exclusively granted to the owner without intermediaries, e.g., without system administrators of hospital, regional, or national information systems. Consequently, the owner can manage their data much like any other private property, though they must acknowledge specific distinctive characteristics inherent to data compared to physical assets.

## 5 Analysis and discussions

### 5.1 Related works

The proposed DCAS-based architecture for personal health data presents an innovative approach to data management, emphasizing user control and data sharing. It resolves three critical health data challenges: accessibility, comprehensiveness, and ownership. In light of these challenges, we outline several initiatives that tackle similar issues.

**MyData global** (77) is a community advocating for human-centric data management, emphasizing data portability, interoperability, and user empowerment. They declare that they “*help people and organizations to benefit from personal data in a human-centric way*.” MyData aims to transform the data economy by ensuring individuals have more control over their data and can share it between services.

**The International Data Space (IDS)** (78) promotes data ownership through its data sovereignty principles, ensuring providers retain control over their data. This framework supports ownership rights across various industries, including healthcare. However, implementing ownership principles within IDS depends on the specific use cases and sectors involved.

**Mediceus** (79) ensures data ownership by providing a user-centric platform where individuals control their health data. Users can manage and share their data securely, maintaining ownership and control. While similar to DCAS in focusing on health data, Mediceus uses a more centralized approach to data management.

**MIDATA's cooperative** (80) model ensures that users are co-owners of their health data. This model prioritizes user interests and provides ownership rights through consent-based data sharing. Users have significant control over their data, although the cooperative model requires active participation and trust in its management.

**Solid project** (81) empowers users with ownership of their data by storing it in Pods (personal data spaces) managed by pod providers. Users can decide who accesses their data and revoke access anytime, ensuring solid data ownership. However, the ownership model is broader and not exclusive.

While these projects address issues related to accessibility, comprehensiveness, and ownership, they fall short of providing a holistic solution to all three.

### 5.2 Interoperability and privacy aspects

As illustrated in Figure 2, according to the proposed reference architecture, every citizen has a personal data space on the DCAS network, where health data as a PHR is preserved under the person's ownership and complete control. A detailed explanation of how health data is represented as PHRs on the DCAS network is beyond the scope of this document. However, we are working toward a unified clinical data model, formalized as RDF-based Knowledge Graphs, which supports ContSys ontology and federated semantic interoperability (66, 67, 82–94).

**Figure 11:**
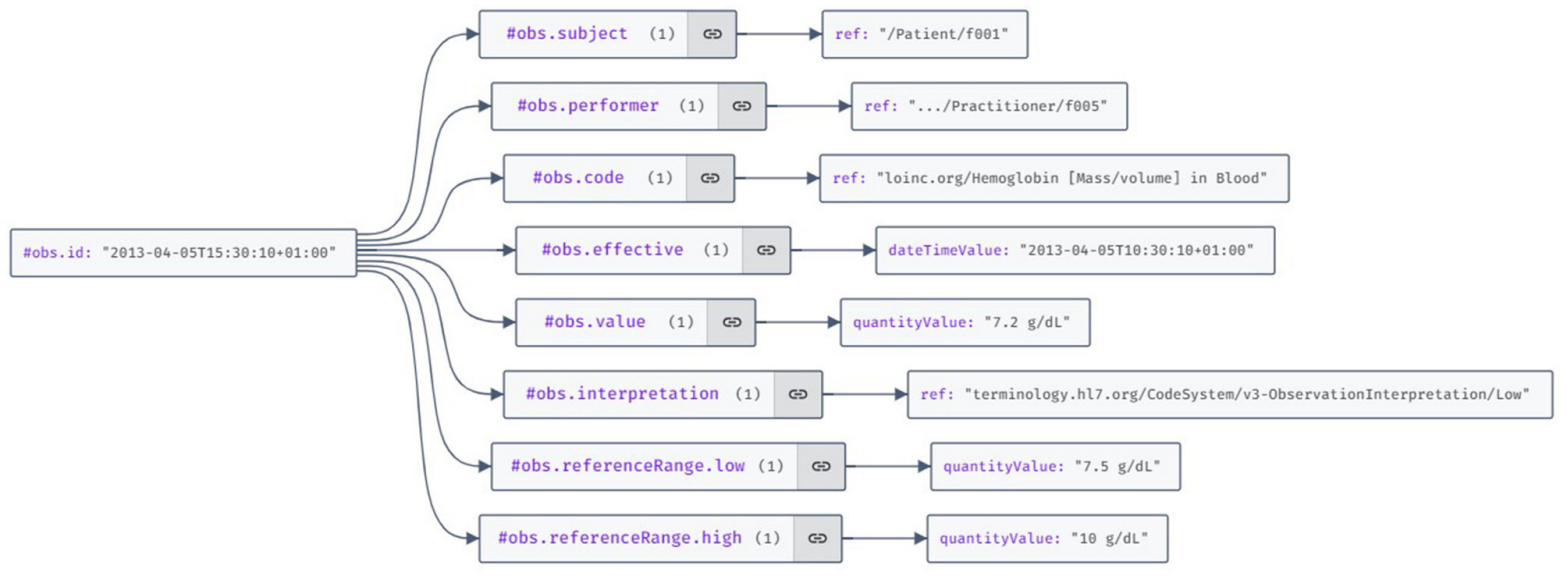
Visualization of the HL7 FHIR (Fast Healthcare Interoperability Resources) observation resource example using the JSON Crack features.

RDF is the standard data interchange model on the Web (95). An FHIR observation resource represented as RDF triplets is illustrated in Figure 11.

Traditionally, the RDF specification employs URIs to represent resources. However, within the realm of DCAS networks, an intriguing prospect arises: substituting URIs with hash values. Such an approach could alleviate numerous issues inherent in URIs, including collisions (distinct resources have the same URL) and aliases (multiple URLs refer to the same resource). By comparing URIs symbol by symbol, a match would unequivocally denote the same resource, eliminating ambiguity. Thanks to the deduplication feature of DCAS networks, it is ensured that a resource cannot possess disparate URIs.

Moreover, the immutable nature of addresses in DCAS guarantees that the meaning associated with any DCAS address-based URI remains constant over time. Unlike URLs on the internet, changes in ownership, and potential unavailability, the hash values (content addresses) of resources on a DCAS network remain unchangeable. This could pave the way for a new version of the internet, aligning closely with Tim Berners-Lee's vision of the Giant Global Graph (96).

We wish to underscore some considerations concerning data de-identification. Firstly, standard FHIR resources conventionally reference the treating physician and the data owner, typically the patient. While usually needed in API requests, this reference becomes redundant when storing data as Personal Health Records in the Personal Knowledge Graph. A more efficient approach involves preserving all demographic data in a distinct data subgraph. An affiliation to the owner is implicitly established by graph connectivity, obviating the explicit need for references to the subject within the resources. This omission of direct references to the data subject streamlines the pseudonymization process, requiring only the sharing of the address of the subgraph housing clinical data. Other identifiable data, such as the treating physician's name and their working institution, can also be separated by preserving them in a separate sub-graph, thus further strengthening the mechanisms for protecting personal data.

Under ordinary circumstances, the root hash of personal data is known exclusively to the data owner. While the owner may share it for primary use by medical service providers, it is conceivable to design protocols facilitating data sharing without divulging the hash. However, for secondary use, a prerequisite is the pseudonymization of the data. This involves creating a pseudonymized copy by expunging all references to individuals, institutions, locations, etc., retaining only essential clinical data. Additionally, all dates within the dataset could be rendered relative to the owner's birthdate. To fortify re-identification control, the hash of the pseudonymized dataset may be integrated into the original dataset, ensuring that only the original owner can reverse the pseudonymization process.

### 5.3 Compatibility with the European Health Data Space

The proposed reference architecture seamlessly aligns with and fully embraces the core principles of the European Health Data Space (EHDS) initiative, offering several valuable enhancements. The following outlines and provides commentary on enhancements resulting directly from the DCAS network characteristics or the proposed reference architecture.

***Data security***. The EHDS advocates for the availability of PHR data via access points established by member states. However, such access points entail heightened data leakage risks. In contrast, the proposed reference architecture employs a DCAS network for storing personal data, significantly mitigating such risks. By decentralizing data access, any potential breach would, at worst, result in the leakage of only one person's data without any impact on the security of others. This minimizes the vulnerability associated with centralized databases, where a breach could compromise millions of individuals' data.

The protocol design achieves data security in a DCAS network. Each network node stores the data locally as a key-value pair. The value corresponds to a distinct data fragment, while the key signifies its address (hash value). Individual fragments are encrypted utilizing distinct keys, rendering the data incomprehensible to the node. Consequently, the network nodes lack access to meaningful information regarding the content of the stored data. Moreover, the network routing protocol ensures that the transmission source of a particular data fragment holds no implications regarding its ownership. In other words, the recipient node remains unaware of whether the sender serves as the original data source or simply functions as an intermediary forwarder. Collectively, these measures signify that network nodes possess no discernible knowledge regarding the content or the rightful owner of the stored data. Consequently, the risk of data leakage becomes virtually negligible within such a system.

The inherently distributed nature of the DCAS network renders it challenging to launch cyber-attacks against it successfully. The absence of a single point of failure confers a substantial advantage, as the network remains unaffected even if specific nodes are compromised due to such attacks. Thus, in theory, the proposed architecture exhibits exceptional resilience against cyber threats.

***Cost efficiency***. Retaining personal data within DCAS networks external to the EHDS infrastructure generates substantial cost reductions for the entire system. This cost-effectiveness stems from two key factors: First, the absence of concentrated personal data in the system eliminates the need for extensive security measures associated with centralized storage and data-sharing protocols. Consequently, the security mechanisms implemented are notably more economical. Second, the utilization of DCAS networks predominantly leverages existing IT infrastructure. This strategic approach significantly diminishes the initial investments required to implement the entire solution and the ongoing expenses essential for its maintenance. The result is a streamlined, cost-effective system that aligns with contemporary economic considerations while ensuring enhanced data security.

***Eliminating single points of failure***. Another vulnerability of storing personal health data in a centralized repository lies in a single point of failure. In centralized repositories, the imperative becomes ensuring regular backups, consequently escalating the overall system cost. In contrast, in DCAS networks, each data point is dispersed across multiple nodes according to the built-in redundancy measures, eliminating the data loss risks associated with a centralized repository. This inherent resilience safeguards against potential data loss and obviates the need for recurrent and resource-intensive backup procedures. Opting for DCAS networks enhances data security and presents a cost-efficient alternative by eradicating the expenses of mitigating the risks of a single point of failure.

***Simplicity***. Eliminating the need to store personal data within central repositories simplifies the system considerably. Typically, an escalation in the complexity of information systems correlates with an augmented security risk, as a more intricate structure expands the potential attack surface (97). A simplified system streamlines operational aspects and inherently mitigates security risks. The logic is straightforward: the less intricate the system, the more manageable and controllable potential security risks become. Simplicity, in this context, acts as a strategic ally, making the system more dependable (98) and security management simpler. Simplicity enhances the system's efficiency and bolsters its security.

***Reducing ecological impact***. Managing health data for hundreds of millions of individuals in centralized systems demands substantial resources, encompassing hardware, energy, and labor, resulting in a notable ecological footprint. A centralized system's infrastructure, by its very nature, has enormous environmental impact. In contrast, DCAS networks utilize resources more efficiently. Operating predominantly on existing infrastructure, they demand relatively few additional resources. Consequently, the ecological footprint of such a decentralized solution is markedly smaller. Utilizing DCAS networks, we enhance the operational efficiency of health data management along with environmental sustainability by making informed choices to minimize the overall ecological impact of health data management systems.

***Empowering data ownership***. The core strategic objective of the EHDS is that of data owners maintaining absolute control over their data. When personal data resides on third-party servers, achieving data owner control becomes challenging. However, adopting DCAS networks establishes a paradigm where data owners have complete and exclusive control over their data. Furthermore, the authority to decide on data sharing rests solely with the owner, reinforcing the realization of the stipulated strategic goal. By embracing DCAS networks, we align with the EU's vision of robust data ownership and establish a framework that empowers individuals with unequivocal access control, ensuring the integrity and privacy of their data per EU strategic objectives.

***Data integrity and version control***. In DCAS networks, utilizing hash values as data addresses guarantees data integrity. Users can compute and compare the hash value with the original data address. A congruence between the two assures the downloader that the downloaded data has not been altered. Furthermore, content addressability introduces an automatic versioning mechanisms' any alteration to the data results in assigning a new address reflective of the modified content. Simultaneously, the prior version of the data persists at its original address. This inherent version control facilitates the preservation of the data modification history. Notably, this characteristic empowers the creation of diverse sub-branches within the data, a useful feature for scenarios requiring selective information disclosure. Subsequently, these branches can be seamlessly amalgamated into a cohesive whole when needed.

***Data preservation***. Given the absence of a central control mechanism, the primary concern within a DCAS network is the preservation of stored data. Volunteers, the main operators of DCAS network nodes, may depart from the network independently. To mitigate the risk of data loss, the network must incorporate effective preservation mechanisms. One such mechanism involves providing rewards to network node operators, which incentivizes them to keep their network nodes online. Additionally, data preservation is facilitated by redundancy, wherein data is distributed across multiple network nodes. Consequently, the departure of a single node does not result in data loss. Ensuring an expansive network size, minimizing the likelihood of node departure, and maintaining sufficient data redundancy make it possible to minimize the probability of data loss to nearly negligible levels.

Re-centralization poses a significant risk to decentralized data networks, referring to accumulating a significant proportion of the network nodes under the control of a single operator. This consolidation empowers the operator to disrupt or halt the network's functionality. To avert this potential threat, the network must attain a substantial scale to render the concentration of a majority of network nodes under the oversight of a single operator unfeasible, both from a technical and financial standpoint. Ensuring a sizable network diminishes the likelihood of re-centralization, safeguarding the network's integrity and resilience.

***Data quality enhancement***. The reference architecture we propose substantially improves data quality. By storing PHR in a single logical location in a unified and coherent manner, issues arising from incomplete or conflicting information can be mitigated by the data owner's validation. Furthermore, the inherent characteristics of DCAS networks automatically guarantee data integrity and facilitate the preservation of a full version history.

***Comprehensiveness***. Storing a PHR in a unified location under the data owner's complete control resolves the prevalent issue of fragmented and incomplete data. Such data completeness effectively tackles the drawbacks associated with the secondary use of health data, which often necessitates gathering data from disparate service providers and increases the data privacy risks associated with secondary use.

***Global scalability***. DCAS networks operate using the Kademlia metric, eliminating the geographical dimension. For redundancy purposes, each data chunk is stored on all nodes belonging to a Kademlia neighborhood. It is important to recognize that within the Kademlia metric, nodes belonging to the same neighborhood may be widely dispersed geographically. In light of this, since each node only stores a small portion of the data, the question of where the data is stored in a geographical sense becomes meaningless. Ultimately, the data is stored simultaneously nowhere and everywhere.

***Data de-duplication***. Within the network, only one logical copy of identical data exists at any given time. This becomes particularly evident when dealing with large, immutable data entities (e.g., images, videos). Even if these entities are included in multiple data sets, such as in the pseudonymization process, only a single logical copy is present within the network. Thus, there is no need for redundant copies of these large data entities; a mere reference to them is sufficient.

### 5.4 Future work

This paper concludes the first part of our research by proposing the reference architecture for resolving health data accessibility, comprehensiveness, and ownership dilemmas by preserving semantically interoperable PHRs in DCAS networks. We have sketched the ideas (99) and submitted the technical solution as an EU patent application (100). Still, we have only proposed a technical solution. The proposed architecture's social, organizational, and legal aspects and applicability in real-life primary and secondary cases are for future study. The same is related to formal and real-life-based evaluation of the properties of DCAS networks in medical, medical emergency, secondary, and private use cases. Therefore, most of the research topics we proposed in Klementi et al. (99) are still to be studied and analyzed. Those topics are as follows:

- *Data model*—currently, we have only preliminary ideas of how the data in PHR in a DCAS network should be preserved; therefore, a data model that supports federated semantic interoperability with the existing and future developed hospital, regional, and national systems and also supports various data communication protocols (e.g., HL7 v.2.7, CDA or FHIR), reference models (e.g., HL7 RIM or openEHR RM), classifiers (SNOMED, ICD, LOINC or their different versions), languages (e.g., English, Estonian) as well as structured and unstructured data must be designed and implemented.
- *Data quality*—the mechanisms must be implemented for how the data is validated technically and clinically before being preserved in PHR in a DCAS network.
- *Data interoperability*—our research group is related to the development of TermX,^1^ a platform for developing healthcare terminology and interoperability and other federated semantic interoperability-related development activities (66, 67, 90, 91, 101).
- *Primary use*—together with physicians, we are designing primary use-case studies to combine real-world clinical and patient-entered data in the treatment of selected diseases, e.g., cardiovascular and prostate diseases.
- *Secondary use*—we are designing different real-world secondary use cases related to clinical trials, public health, medical statistics, care efficiency, quality, etc.
- *Data security and privacy*—one of the directions here is to design a technical and organizational solution for health data de-identification so that the de-identified data is reliable for secondary use; another direction is to design and conduct proper real-world evidence-based experiments to justify these properties in primary and secondary use-cases.
- *Data integrity and transparency*—although data integrity and transparency arise from DCAS properties, we have to justify these in real-world evidence-based experiments during primary and secondary use.
- *Linked data*—the potential role of a DCAS network as the foundation for the Giant Global Graph (by Tim Berners-Lee) is an interesting related research topic.

## 6 Conclusion

The reuse of health data presents a significant challenge that currently lacks an effective solution. This article delves into the issue through the lenses of accessibility, completeness, and ownership. To address these challenges, we propose a novel, globally scalable architecture for a personal health data space based on decentralized content-addressable networks. It ensures that data subjects retain complete and exclusive control over their data, while enabling them to share it with third parties as they see fit.

To illustrate the problems, we present four use cases from the Estonian e-health system, demonstrating how the current methods fail to effectively address the three dilemmas. Following this, we analyze how the proposed new strategy resolves these issues.

The proposed architecture presents a notable departure from previous approaches to health data management and introduces a paradigm shift in the manner in which data storage is conceived. Therefore, it is expected that society will require a significant period of adjustment. Consequently, the feasibility of implementing the described solution in the immediate future appears remote. Nonetheless, it remains imperative for societal discourse to acclimate to emerging technological possibilities and navigate alongside them.

By providing enhanced control, interoperability, security, and transparency, the proposed solution has the potential to fundamentally transform how individuals interact with their health data. It empowers individuals to take an active role in their healthcare journey, fostering a more patient-centric and secure healthcare environment.

## Data availability statement

The original contributions presented in the study are included in the article/supplementary material, further inquiries can be directed to the corresponding author.

## Author contributions

TK: Conceptualization, Investigation, Software, Writing – original draft, Writing – review & editing. GP: Funding acquisition, Investigation, Methodology, Resources, Software, Supervision, Writing – original draft, Writing – review & editing. PR: Funding acquisition, Investigation, Methodology, Project administration, Resources, Supervision, Writing – original draft, Writing – review & editing.

## Footnotes

^1^ https://termx.org/
